# Bluetooth-Based Dynamic Nexus Mesh Communication Network for High-Density Urban Interaction Spaces

**DOI:** 10.3390/s25082495

**Published:** 2025-04-15

**Authors:** Yufei Hu, Ngai Cheong, Muya Yao, Qingwen Long, Yide Yu

**Affiliations:** 1 Faculty of Applied Sciences, Macao Polytechnic University, Macau 999078, China; p2311715@mpu.edu.mo (Y.H.); p2312044@mpu.edu.mo (M.Y.); yide.yu@mpu.edu.mo (Y.Y.); 2 School of Intelligent Transportation Engineering, Guangdong Communication Polytechnic, Guangzhou 510650, China; longqw@gdcp.edu.cn

**Keywords:** DNMC network, real-time interaction, high-density urban interaction spaces

## Abstract

Traditional centralized network structures exhibit clear scalability and communication efficiency bottlenecks. This paper proposes a solution based on a bidirectional unweighted heterogeneous graph, Dynamic Nexus Mesh Communication, designed to improve communication efficiency and optimize user experience, particularly for high-density urban interaction spaces. DNMC reduces the reliance on central super nodes in traditional centralized networks by redistributing network centrality while increasing the betweenness centrality of a broader set of nodes. Additionally, DNMC introduces multiple node types, improving both the robustness and efficiency of the network. Through a series of simulation experiments, we compared the communication performance of DNMC networks with that of traditional centralized networks. The results show that DNMC achieves an average delay of 1.5824 s, representing a 13.69% improvement over centralized architectures. These findings demonstrate that DNMC significantly outperforms traditional networks in terms of communication efficiency, particularly at larger network scales, where DNMC exhibits enhanced stability and scalability.

## 1. Introduction

As urbanization continues to accelerate and the demand for intelligent management increases, global interaction patterns and information flows are experiencing significant transformations. The growing interconnectedness of cities presents considerable challenges to the traditional centralized network model, which often fails to meet the demands of modern urban infrastructure. The shortcomings of centralized systems—in particular, their scalability, flexibility, and responsiveness—become even more pronounced in high-density urban settings. In these environments, managing and scheduling resources, data processing, and security in large public spaces, transportation hubs, and entertainment centers are essential and require real-time, precise solutions.

Traditional centralized communication systems rely on central servers for data transmission and resource allocation, creating risks of single points of failure and limiting scalability. As user and device numbers grow, these systems face challenges like increased communication delays and reduced efficiency, especially in densely populated urban areas. Decentralized communication models, particularly peer-to-peer architectures, offer distinct advantages. Additionally, techniques like Multidimensional Index Modulation Differential Chaos Shift Keying have shown potential in enhancing the data rate and energy efficiency for low-power, short-range wireless communication [1,2]. While decentralized solutions leveraging the Internet of Things and edge computing have advanced in smart city applications, they still face significant hurdles. Network stability can be disrupted by environmental factors, requiring adaptation to frequent topology changes. As systems scale, ensuring efficient collaboration and resource management becomes crucial in high-density urban environments.

To address these issues, this paper proposes a new decentralized communication architecture, the Dynamic Nexus Mesh Communication (DNMC) network, which aims to overcome the limitations of existing centralized and distributed network systems in high-density urban interactive spaces. In this study, ‘high density’ is characterized in two dimensions: external high density and internal high density. External high density refers to the concentration of population and infrastructure, exemplified by closely spaced supermarkets and parking facilities in urban environments. These settings create frequent interactions and high communication demands, making them well-suited for DNMC deployment. Internal high density describes the dense distribution of network nodes within DNMC, which enhances network resilience and efficiency through optimized multi-hop routing and seamless connectivity in dynamic environments. By integrating both dimensions, DNMC is designed to support large-scale, high-interaction urban spaces, addressing scalability and adaptability challenges that traditional centralized networks may struggle to overcome.

DNMC builds a dynamic mesh network based on Bluetooth technology containing multiple node types (user, information source, functional module, and routing nodes). By redistributing the network’s centrality and enhancing the intermediary centrality between nodes, DNMC effectively improves the network’s communication efficiency and robustness. Its decentralized characteristics make data transmission and resource allocation more flexible and efficient, and it is particularly suitable for complex urban environments that require high scalability, reliability, and low-latency communications.

The following are the key contributions of this work:

(1) The introduction of an innovative DNMC network architecture: We have implemented a DNMC network that utilizes Bluetooth technology to interconnect different types of nodes, such as user nodes, information source nodes, functional module nodes, and routing nodes. This architecture notably improves the system’s flexibility and reliability, demonstrating its distinct advantages in meeting the intricate requirements of managing high-density urban interaction spaces.

(2) Validation of the DNMC network’s performance: We performed a series of thorough simulations to compare the DNMC network with traditional centralized network architectures. The results showed that the DNMC network consistently performs better than conventional systems in terms of communication efficiency and delay.

## 2. Literature Review

As the demand for intelligent city management and high-density interaction spaces continues to grow, research into the effective management and optimization of resources in high-density urban spaces provides new insights. Integrated communication methods are often used to manage large venues and streamline data processing and equipment management [3,4]. For instance, Guo et al. examined centralized control and management systems for large-scale venues [5,6,7]. Biyik et al. delved into technical aspects of intelligent parking solutions, including system architecture, sensor integration, and communication protocols [8,9]. Wang proposed an indoor car-finding and navigation system based on Bluetooth low-energy (BLE) technology, which mainly solves the problem of finding cars and pedestrian navigation in parking lots. The system uses BLE technology to achieve more efficient indoor positioning and navigation, which is especially suitable for closed spaces such as parking lots [10]. Environmental factors, such as weather changes, can also impact the performance of sensor-based systems [11]. Qamas et al. proposed a cloud-based Smart Vehicle Parking System where parking management is conducted via a centralized cloud server. However, this approach encounters challenges such as high hardware costs, limited system versatility and privacy concerns, and slow efficiency in storing and processing large amounts of data on cloud servers [12,13,14,15,16].

In exploring intelligent solutions, collaborative communication models optimize resource usage and data transmission efficiency through coordination among multiple nodes [17,18,19]. Arellano et al. developed a simulation based on the Veins framework to evaluate the algorithm’s performance. They proposed a new multi-hop, decentralized, and colony-inspired dynamic highway traffic assignment algorithm that is suitable for the current traffic congestion situation of large road networks [20]. Sifakis et al. introduced a blockchain-based multi-agent system for real-time power management in a virtual producer–seller network, which can optimize geographically distributed scheduling and coordination [21]. Mane et al. have introduced an IoT-based intelligent parking system that utilizes a distributed communication approach to optimize parking space utilization by implementing time resource sharing, enabling the early identification and reservation of parking spaces [22,23,24]. Similarly, Mouhcine et al. proposed a distributed IoT-based smart parking guidance system [25,26]. Moreover, the substantial initial investment and ongoing maintenance costs present significant financial challenges that may impact the system’s long-term sustainability [27,28].

Contemporary advancements in intelligent management systems, emphasizing decentralized communication methodologies, particularly through peer-to-peer networks and edge computing, significantly propel the trajectory toward achieving heightened system adaptability and scalability levels [29,30,31,32]. Li et al. delineate an architecture for smart parking systems predicated upon the foundations of P2P networks coupled with edge computing paradigms [33]. In traditional networks, degree centrality is used to identify highly connected nodes; however, in dynamic urban environments, relying solely on nodes with high degrees can lead to bottlenecks and create single points of failure. In contrast, betweenness centrality measures how often a node lies on the shortest paths among others, making it essential for maintaining efficient communication, especially when nodes frequently join or leave the network. By optimizing betweenness centrality, the DNMC network ensures a decentralized flow of data, reducing dependence on specific nodes and enhancing resilience against interference or node failures. This approach improves fault tolerance and load balancing, thereby ensuring stable and efficient communication in large-scale, high-density settings.

This paper presents a novel dynamic nexus mesh communication network based on Bluetooth. Our designed network effectively manages flow control functions across various scenarios while requiring minimal programming. It consists of multiple low-cost microcontrollers. This solution is applicable in several settings, including large parking lots, convention centers, and performance venues.

## 3. Proposed Solutions and Design

### 3.1. Overview of the Overall Design

**Figure 1:**
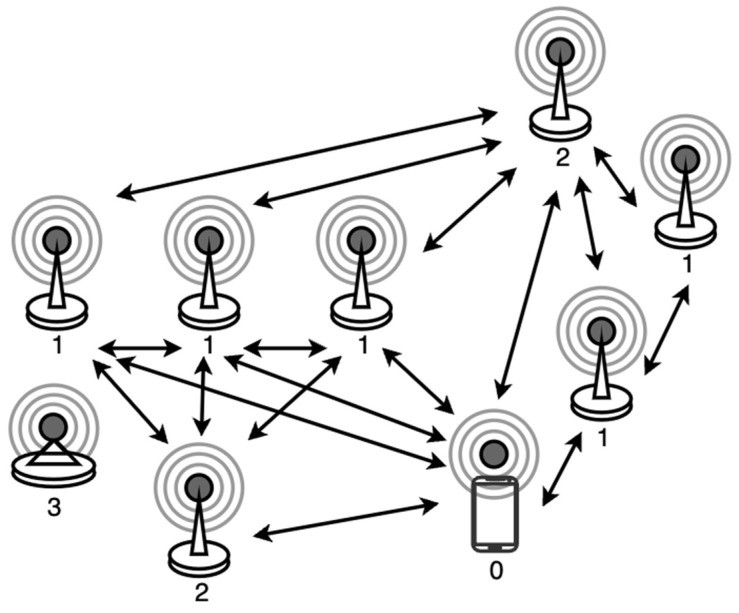
Fundamental architecture of the DNMC network.

The proposed design leverages Bluetooth technology to establish a DNMC network of various peer nodes. These nodes encompass user nodes, information source nodes, functional module nodes, and more. The transmission of network signals is facilitated and optimized by routing nodes to ensure efficient data delivery within high-density urban interaction spaces such as parking lots and concert arenas. For example, information source nodes, like sensor nodes, are responsible for real-time monitoring and updating parking space occupancy in a parking lot scenario. Subsequently, these updates are disseminated to other nodes within the network. Functional module nodes, such as display devices and LED indicators, utilize the received information to offer real-time navigation and visual guidance. This enables users to swiftly locate available parking spaces and enhance the overall parking experience. The diagram in Figure 1 depicts the fundamental architecture of the DNMC network. Node 0 corresponds to user nodes, Node 1 corresponds to information source nodes, Node 2 corresponds to functional module nodes, and Node 3 corresponds to routing nodes. These four categories of peer nodes establish a small-scale peer-to-peer network ecosystem, facilitating peer-to-peer interaction without reliance on a central server. Expanding this network would extend the potential application of this design to larger-scale scenarios.

Mathematically, the DNMC network is represented as a bidirectional unweighted heterogeneous graph $G=\left(V,\;ℇ,\;T\right)$, where V denotes the set of nodes, ℇ denotes the set of edges, T is the set of node types, with $T=\{T_{1},T_{2},T_{3}\}$, where each $T_{i}$ represents a distinct type of node.

In graph G, the function for node mappings’ node-type relations is ∅: V → T. This graph is strongly connected because for any ‘u, v ∈V, $\left(u\neq v\right)\Rightarrow \left(\mathrm{uv}\;\wedge \;\mathrm{vu}\right)’$, where uv means a directed path from node u to node v, and vu is a directed path from node v to node u.

The upper bound of the unidirectional shortest path for connecting the user and terminal equipment in graph $G\;\mathrm{is}\;3+|T_{2}|$, where 3 contains a user node, an information source node, and a function module node. $|T_{2}|$ is the cardinality of the type node set of the DNMC network and routing nodes. According to Breadth-First Search, the time complexity for finding the shortest path is $\vartheta \left(V+ℇ\right)$. 

In the traditional network architecture $G^{\prime }=\left(V^{\prime },E^{\prime },T^{\prime }\right)$, the centralized node (super node $v^{*}$) has high degree centrality, which is formed as

$$\forall v^{\prime },\;{v^{\prime }}^{*}\in V^{\prime },\;CD\left({v^{\prime }}^{*}\right)>\;C_{D}\left(v^{\prime }\right)\Rightarrow \frac{\deg\left({v^{\prime }}^{*}\right)}{n-1}>\frac{\deg\left(v^{\prime }\right)}{n-1},$$

where $\deg\left(v\right)$ is the degree of node v, n is the cardinality of the node set ∣V∣, and $C_{D}\left(v\right)$ is the degree centrality. However, in the DNMC network, the centralized node reduces the degree of centrality and improves the betweenness centrality, which is expressed as follows:

$${\forall v^{*}\in V,{v\prime }^{*}\in V^{\prime },C}_{D}\left({v\prime }^{*}\right)>\;C_{D}\left(v^{*}\right)\mathrm{and}\;C_{B}\left({v\prime }^{*}\right)<\;C_{B}\left(v^{*}\right),$$

where the $C_{B}\left(v\right)$ is the betweenness centrality of the node v. In general, the betweenness centrality is

$$C_{B}\left(v\right)=\sum_{s\neq v\neq t}\frac{\sigma_{\mathrm{st}}\left(v\right)}{\sigma_{\mathrm{st}}},$$

where $\sigma_{\mathrm{st}}$ is the number of shortest paths from the source node s to target node t, and $\sigma_{\mathrm{st}}\left(v\right)$ is the number of paths from node s to node t that pass through node v in the shortest path.

The traditional method of obtaining location and exit information in high-density urban interaction spaces such as parking lots, shopping centers, and concert arenas relies on staff conducting on-site observations and using their experience. Based on the current situation, staff typically provide path guidance and select optimal locations or exits. However, our design utilizes Bluetooth technology to establish a DNMC peer-to-peer network that covers all information and user nodes, thus enabling intelligent venue management.

**Figure 2:**
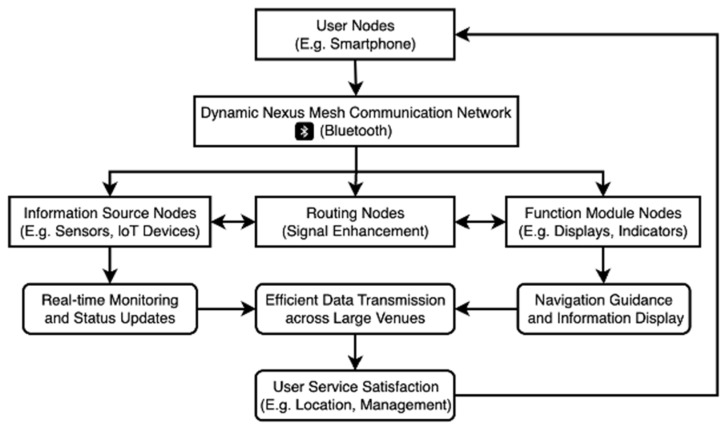
Workflow diagram of DNMC network.

In this network, user nodes, like smartphones, connect to the DNMC network within a venue and are assigned unique user identifiers, such as user001, user002, and so forth. When a user requests navigation to a specific target location—be it a parking spot near a supermarket or cinema, a concert seat, or another location tailored to their needs—the system leverages sensor nodes distributed throughout the venue to monitor the status of various areas in real time. These sensor nodes are equipped with devices like distance and weight sensors, which continuously update the occupancy status of each location and relay this information to the main nodes within the venue. Figure 2 provides a workflow diagram of the DNMC network.

The primary nodes within the system operate similarly to traditional control modules, but they are enhanced with Bluetooth communication capabilities, integrated computing units, and local storage. These nodes process data from sensor nodes, optimize user location allocation, and determine the most efficient navigation paths. Display screens are strategically positioned at key intersections or nodes within the venue to present user identifiers along with navigation directions, facilitating the easy identification of intended locations. Additionally, when users are prepared to leave the venue, the system swiftly calculates and provides guidance for the most efficient exit route in real-time, ensuring prompt departures. The DNMC design of the entire system enhances the reliability of information transmission while also improving flexibility and scalability, making it ideally suited for the intelligent management needs of various venues.

### 3.2. Components and Implementation of DNMC Network

To cater to the sophisticated management needs of expansive venues, we have established user nodes, DNMC network and routing nodes, information source nodes, and function module nodes as part of the network design. This approach ensures the system’s dependability, adaptability, and enhanced user experience. Below is a detailed overview of the components and implementation of this design.

#### 3.2.1. User Nodes

In contrast to the relatively fixed nature of traditional desktop computers, smartphones have gained widespread adoption on a large scale and are the foundational devices for utilizing Bluetooth technology. Given their significant popularity and users’ familiarity with them, we have opted to use smartphones as user nodes. In addition, for the high-density urban interaction spaces, we can provide a small module to each user to facilitate communication and convey user needs. Our choice focuses on enabling mobility and convenience for user nodes, allowing them to seamlessly connect to various networks of different scales and functions during communication without additional hardware. This approach significantly reduces the system’s deployment costs. Whether utilizing smartphones or small, expandable integrated modules, they can support multiple communication protocols and sensing technologies, enabling seamless integration with other nodes in the system (such as information source nodes and function module nodes) to achieve efficient data interaction and service access. This enables users to access personalized smartphone services, such as path guidance.

#### 3.2.2. Routing Nodes

While traditional networks often do not require routing nodes, ensuring signal coverage and transmission stability is paramount in many large and structurally complex venues. Therefore, in our network architecture design, we strategically deployed routing nodes to enhance signal coverage, ensuring consistent reception throughout the venue. Furthermore, deploying redundant routing nodes enables the network to sustain a stable operation even in the event of inevitable node failures. This strategic choice focuses on enhancing signal transmission and adjusting signal paths to accommodate various environmental changes.

#### 3.2.3. Information Source Nodes

In designing this network, we aim to create a specific type of peer node dedicated to the real-time acquisition of diverse environmental information, known as information source nodes. Common examples of information source nodes include sensors and IoT devices. These nodes can directly engage in the overall network, ensuring the continuous and reliable provision of real-time data. These data can be swiftly transmitted to other network nodes via Bluetooth technology. For example, in the context of parking lots, these nodes can accurately detect parking space occupancy and monitor road traffic conditions. Similarly, in a concert setting, they can determine seating arrangements and occupancy, while in traffic management applications, they can track vehicle movement and gather crucial information at intersections.

#### 3.2.4. Function Module Nodes

Function module nodes are specifically designed to cater to the personalized needs of users within high-density urban interaction spaces, and the quality of their experience relies heavily on these nodes. This section has established various common functional module nodes, such as display devices and LED indicators. These nodes are crucial as they can offer users the necessary information in a clear and real-time manner, enabling them to locate their destinations more efficiently and conveniently. By using display screens and LED indicators, users can easily identify their unique identifier and receive accurate navigation guidance, thereby reducing confusion and disorientation within the venue. Moreover, data from information source nodes can be instantly relayed to the function module nodes, ensuring that the displayed information is always current. Additionally, the content displayed by the function module nodes can be tailored as needed to accommodate different scenarios and usage requirements.

### 3.3. Communication Protocol Design

**Figure 3:**
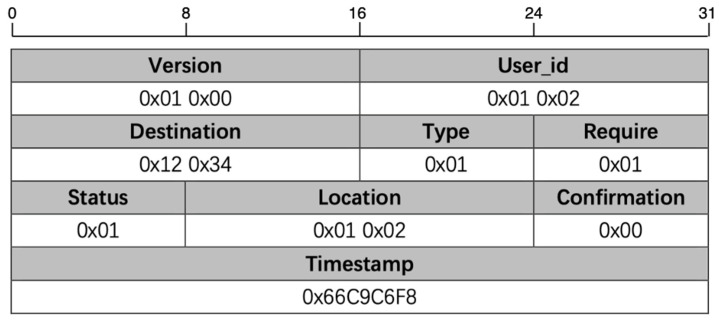
Communication protocol design.

To address the demands of efficient management in today’s expansive venues, this communication protocol employs unique user identifiers and real-time data interactions to effectively allocate resources and enhance the user experience. The protocol’s design carefully balances reliability, flexibility, and scalability, making it particularly well-suited to the diverse management needs of high-density urban interaction spaces. Figure 3 provides an overview of the communication protocol within this framework.

The <Version> field can be used to view the software version number on which the communication is based. Each communication protocol message packet includes a version field to ensure the compatibility of the message format and the backward compatibility of the system, meeting specific user needs for different version functionalities. The <User_id> field is used to identify the user in the communication. When a user node connects to the DNMC network, it is assigned a unique user identifier (e.g., user001, user002), uniquely identifying the user in the communication. The <Destination> field identifies the signal recipient and is used to send a request to the system to reach a target location, which is represented by multiple identifiers. The <Type> field identifies the type of user request, such as navigation requests, location status inquiries, etc. This is the communication protocol’s core, defining the message’s basic nature and purpose (e.g., status update, content query, user verification), allowing for quick identification and processing of the received data. The <Require> field defines the user’s specific needs, such as a request for help, searching for a resource, or any other form of demand. The <Status> field represents the user’s current status or the status of the request, providing feedback on the status of the current or target location, such as occupied, available, or malfunctioning. The <Location> field can be used to support location-based services, where the system uses this information to provide navigation and location-allocation services. The accuracy of the location information is crucial for delivering personalized services. The <Timestamp> field allows the system to track when events occur and manage time-based reservations, ensuring the real-time nature and consistency of sensor data and user requests. The <Confirmation> field provides a Boolean feedback value indicating the success or failure of a specific operation or request. After the system calculates the optimal path or location for the user, the user can confirm the received guidance information and proceed accordingly.

Through field compression and optimization, we have successfully customized the length of each field in the communication protocol. This design maintains a universal structure and allows for the extension of new fields to accommodate various functional requirements in personalized application scenarios. In this framework, each data segment is limited to a maximum of four bytes, ensuring efficient data transmission while meeting diverse system needs.

Taking a parking operation request as an example, the current protocol version is 1.0, and the user ID (0×01 0×02) represents the vehicle owner requesting to park in a designated area (0×12 0×34), with the operation type set to 0×01 (parking request). The system indicates an available parking slot (status 0×01), while the request has not yet been confirmed (Confirmation field 0×00). The Timestamp and Confirmation fields ensure real-time reservation management, data consistency, and seamless user interaction. When the user submits a reservation request via mobile, the system generates and records a Timestamp (e.g., 0×66C9C6F8), marking the parking slot as “reserved” to prevent conflicts. The system then assigns an appropriate slot and sets a reservation validity period (e.g., 15 min). The slot is automatically released if the user fails to arrive within this period. Once the slot is locked, the system updates the Timestamp (e.g., 0×66C9C700) to ensure traceability. Upon the user’s arrival, vehicle detection or user input triggers the Confirmation mechanism, verifying the slot’s usage. This design facilitates efficient parking management, precise time synchronization, and optimized user interaction, ensuring a real-time, stable, and reliable reservation process.

## 4. Experimental Testing

### 4.1. Scenario Simulation

**Figure 4:**
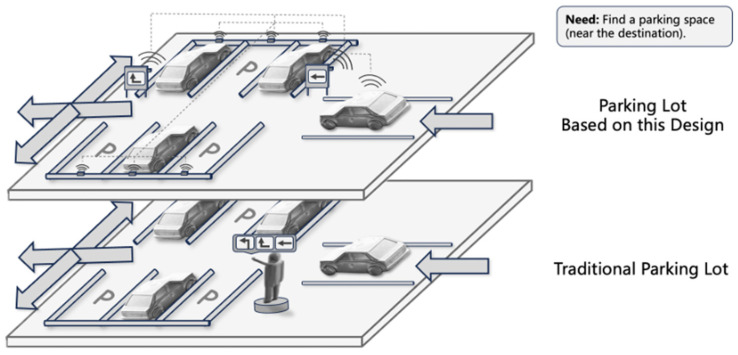
Three-dimensional comparison of different parking lots.

To further validate the proposed concept, we conducted simulation testing using a parking lot as the primary application scenario. Our goal was to confirm whether our DNMC network, based on Bluetooth technology, could improve the efficiency of intelligent management and enhance user experience in high-density urban interaction spaces. Figure 4 compares the modeling between the parking lot using the DNMC network and a traditional parking lot.

**Figure 5:**
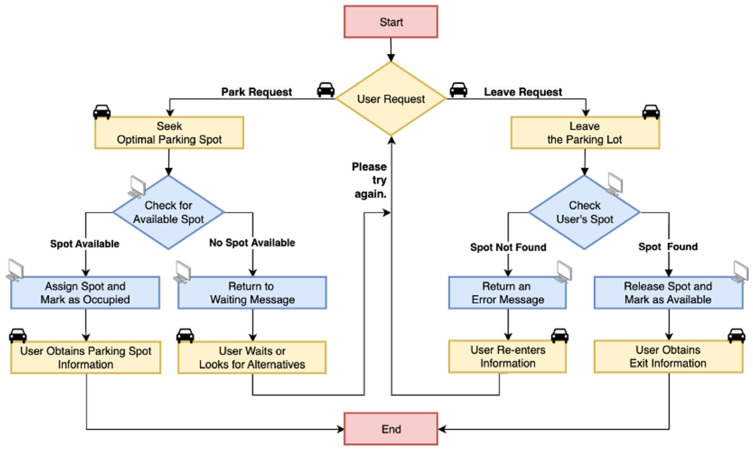
Scenario workflow of a parking lot using the DNMC network.

The workflow diagram in Figure 5 demonstrates the implementation of our design within an intelligent parking lot. The flowchart details two main components of the intelligent parking system: parking requests and exit requests. When a user enters the parking lot, the system begins processing their parking request. It then performs a real-time check for available parking spaces by utilizing parking space sensor nodes to assess occupancy status.

This section will simulate the intelligent parking lot to illustrate what happens when a user enters. There are two possible scenarios:

(a) Spot Available: In this case, the system will assign the user an available parking space and mark it as occupied. The user will then receive a success message confirming the parking space allocation. Display devices at each intersection will guide the vehicle’s direction (left turn, straight, right turn), and an LED indicator will light up in real time at the allocated parking space.

(b) No Spot Available: If there are no available parking spaces available, the system will notify the user to wait. The user can choose to continue waiting for a space or look for alternative options.

Users can initiate an exit request before leaving the parking lot. Upon receiving this request, the system will retrieve the user’s assigned parking space based on their user ID and provide the relevant information. This can result in two scenarios:

(a) Spot Not Found: If the system cannot locate the user’s parking space, it will return an error message, prompting the user to re-enter their parking information or provide additional relevant details to locate the parking spot.

(b) Spot Found: The system will release the user’s parking space and mark it as available within the system. Once the system confirms that the space has been released, it will send a successful exit message to the user. Throughout this process, display screens and LED indicators will offer real-time navigation and visual guidance, including directions to the designated parking space and instructions for exiting the parking lot.

Evidently, this intelligent parking system not only caters to users’ needs but also significantly enhances parking efficiency, reduces labor costs, and ensures the effective and rational management of parking spaces.

### 4.2. Experimental Simulation

This experiment was conducted using Python 3.8 as the simulation environment, integrating multiple tools to implement the model. NetworkX was used for network topology construction, while SimPy was employed for discrete event-driven communication simulation. Additionally, a random number generation mechanism was introduced to model dynamic node connections and communication latency under varying transmission conditions. Both DNMC and traditional centralized networks were constructed in the simulation, with parameter k dynamically adjusting network scale.

**Figure 6:**
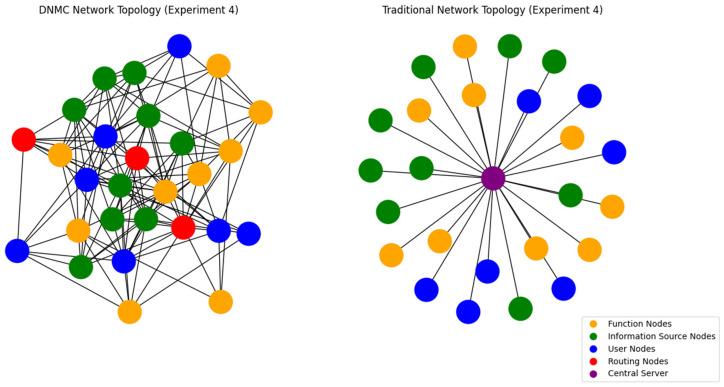
Network topologies of DNMC and traditional network.

**Figure 7:**
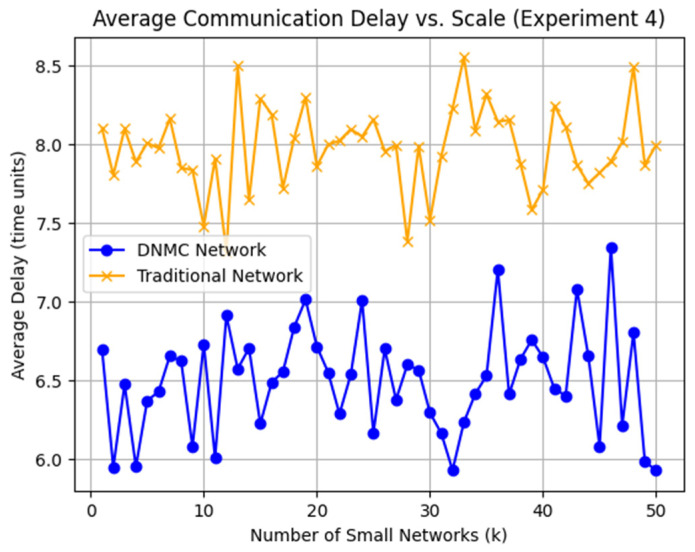
Communication delay comparison between DNMC and traditional network.

The DNMC topology consists of functional module nodes, information source nodes, user nodes, and routing nodes, where functional and information source nodes are randomly distributed within 1 to 4k, while user nodes also vary within this range. Each DNMC network includes k routing nodes responsible for signal amplification and data relay, improving communication efficiency and coverage. Node connections are established using a random connectivity mechanism, where the probability of a connection between any two nodes is set to 0.2, simulating a dynamic topology. In contrast, the traditional centralized network follows a different architecture, where all functional module nodes, information source nodes, and user nodes connect directly to a central server, which serves as the sole communication relay. While stable in structure, this centralized approach limits communication flexibility. To illustrate the differences between these architectures, multiple simulation comparisons were conducted. Figure 6 presents the topological contrast between DNMC and centralized networks, while Figure 7 visualizes latency variations across different network scales.

Multiple nodes were randomly selected for data transmission in both environments, and end-to-end transmission delays were measured. In DNMC, data are transmitted along the shortest available path within the network. In contrast, in centralized networks, all data must first be relayed through the central server, forming a fixed two-hop transmission structure.

To evaluate the communication performance of both network architectures, we analyzed their respective delays under different conditions. This entailed measuring the communication delay between nodes as the network size expanded. We conducted multiple delay measurements for both the DNMC and traditional centralized networks and calculated the average delay for each network using Equation (1).

(1) $$\mathrm{Average\_Delay}=\frac{1}{N}\sum_{i=1}^{N}d_{i}$$

N denotes the number of communication events, and di represents the delay value for each communication. The reported average delay values of 1.5824 for the DNMC network and 1.8333 for the traditional centralized network were obtained by computing the mean delay across multiple independent simulation runs.

Experimental results indicate that DNMC significantly reduces the average communication latency compared to centralized networks, particularly as the scale of the network increases. While DNMC does experience some latency fluctuations due to dynamic conditions, it consistently maintains lower overall delays through the use of multi-hop routing and load-balancing strategies. In contrast, centralized networks exhibit stable yet higher amounts of communication delays, primarily due to the bottleneck effects associated with the central server. These findings underscore DNMC’s suitability for large-scale, high-concurrency environments, such as high-density urban interaction spaces.

### 4.3. Experimental Verification

DNMC is well-suited for high-density urban interaction spaces, including large shopping malls, concert venues, and smart parking facilities—scenarios characterized by high-frequency node usage and complex interactions. To validate DNMC’s adaptability, the experiment incorporated a variable configuration approach, adjusting node deployment density, user access rates, and data exchange frequencies to simulate environments of different scales and complexities. Additionally, we accounted for real-world factors, such as physical obstructions affecting BLE signal propagation, and implemented adaptive multi-hop mechanisms to optimize data transmission paths.

According to the basic network architecture shown in Figure 1, we built a real hardware platform that is consistent with the parking lot scenario in Figure 4. The platform utilizes smartphones as user nodes and features a custom WeChat applet for user interaction.

For the platform routing node based on ESP32 as the kernel of the Bluetooth communication module, the module has a 521 K SRAM on-chip memory, 8 M off-chip memory PSRAM, rich storage space, and sends power in +4 Bm; its Bluetooth networking method BLE mesh transmission distance is up to 100 m. the source of information nodes using ultrasonic parking space sensors, the function of the node to choose the LED display, each region can be configured according to the actual demand if the node can be used for driving instructions; take the K value of 5, and for each module routing node identified as A, B, C, D, or E, where each node has a unique ID value and application, all nodes compose the DNMC network. The user node information is shared with all the needed nodes through an autonomous join-and-exit Bluetooth communication interaction mechanism.

Multiple protection mechanisms were implemented at hardware and network levels to ensure DNMC network security. The ESP32 module integrates an AES-128 encryption engine to secure data transmission, preventing unauthorized access. DNMC employs end-to-end encryption with session keys to ensure secure inter-node communication. A decentralized identity authentication mechanism using a public–private key system verifies user nodes, preventing malicious impersonation. Timestamp and hash chain mechanisms enhance authentication security during dynamic node additions and removals, strengthening the network’s resilience against attacks. DNMC also optimizes energy efficiency through BLE mesh networking, which consumes significantly less power than Wi-Fi or cellular networks. In our experiment, ESP32 routing nodes operated at +4 dBm, achieving a 100 m range while maintaining an average power consumption of 120–150 mW—which is over 50% lower than traditional Wi-Fi solutions. An event-triggered wake-up mechanism ensures that nodes remain inactive when no user requests are detected, reducing overall consumption by 30%. Multi-hop routing optimization further minimizes redundant transmissions, enhancing network efficiency.

**Figure 8:**
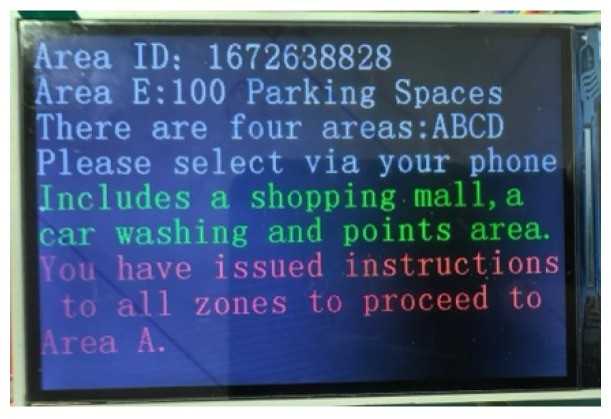
Functional node display effect of the DNMC network.

During the experiment, module E was closest to the user nodes, while A, B, C, and D were distributed in different parking lot areas. Various nodes can join or exit freely after powering up the autonomous network. The user first informs module E through their smartphone that the destination area they want to go to is D. Figure 8 shows the functional node display effect of the DNMC network. After E obtains the user’s information, the remaining four user nodes become aware of the user’s purpose through the DNMC network. Each node synthesizes information from the source node as well as other relevant data from nodes in their respective regions, corresponding to the function node, which is the screen that provides the user with the best driving path; the B region function node guides the car owner to the D area for optimal route guidance. The test of moving distance and reaction time shows that the platform fully meets the user’s parking path demands.

Experimental results demonstrate that DNMC effectively directs users to their desired parking spots by sharing real-time information and autonomous route adjustments. The system facilitates seamless communication between nodes and dynamically updates navigation instructions based on user input and environmental conditions. With its self-adaptive routing and path optimization capabilities, DNMC enhances its robustness in real-world parking situations. These findings validate the feasibility and effectiveness of DNMC in improving parking efficiency and the user experience, presenting a reliable solution for high-density urban interaction spaces.

## 5. Conclusions

This study introduces an innovative DNMC network architecture designed for high-density urban interaction spaces. By leveraging Bluetooth technology, DNMC integrates user, information source, and functional module nodes, enabling real-time data transmission and resource allocation without relying on a central server. Comparative simulations demonstrate that DNMC consistently achieves lower communication delays than traditional centralized networks, especially as the network scale increases. The experimental results highlight DNMC’s resilience and flexibility in addressing complex urban challenges, offering a decentralized, efficient solution for managing high-density environments. Future work will focus on enhancing DNMC by optimizing routing algorithms to reduce latency further and improve load balancing in large-scale deployments. Integrating edge computing could enable local data processing, minimizing reliance on cloud infrastructure, while AI-driven adaptive routing can dynamically optimize transmission paths based on real-time conditions. Beyond parking management and navigation, DNMC has the potential to support intelligent transportation, large-scale event coordination, and smart retail systems, further expanding its applications in dynamic urban ecosystems.

## Data Availability

Data are contained within this article.
